# Clinical effects of cervical conization with positive margins in cervical cancer

**DOI:** 10.1038/s41598-021-02635-y

**Published:** 2021-12-02

**Authors:** Yukari Nagao, Akira Yokoi, Kosuke Yoshida, Masanori Sumi, Masato Yoshihara, Satoshi Tamauchi, Yoshiki Ikeda, Nobuhisa Yoshikawa, Kimihiro Nishino, Kaoru Niimi, Hiroaki Kajiyama

**Affiliations:** 1 Department of Obstetrics and Gynecology, Nagoya University Graduate School of Medicine, Tsuruma-cho 65, Showa-ku, Nagoya, 466-8550 Japan; 2 Institute for Advanced Research, Nagoya University, Nagoya, Japan

**Keywords:** Cancer, Oncology

## Abstract

Radical surgery after cervical conization is a common approach for the treatment of cervical cancer. In some cases, disease progression is observed after positive margins at conization, but the effect of conization on disease progression remains unclear. Thus, the aim of this study was to investigate the clinical outcomes of positive margins at conization in cervical cancer. A total of 101 patients who underwent cervical conization before radical hysterectomy and pelvic lymph node dissection were considered eligible by reviewing medical records. The association between the positive margins and patient outcomes, including subsequent lymph node metastasis, was evaluated. The rate of lymphovascular space invasion (LVSI) positivity at radical surgery was significantly higher in patients with positive margins (*p* = 0.017) than in those with negative margins, although there was no significant difference in the rate of pelvic lymph node metastasis (*p* = 0.155). Moreover, there was no significant difference in the overall survival or progression-free survival between the two groups (*p* = 0.332 and 0.200, respectively). A positive margin at conization presented no significant prognostic disadvantage; thus, diagnostic conization is one of the most suitable treatment options for early-stage cervical cancer that is difficult to accurately assess.

## Introduction

Cervical cancer remains one of the major health problems for women. In 2020, approximately 600,000 patients were diagnosed with cervical cancer globally, and > 340,000 patients died owing to disease progression^1^. In comparison with other developed countries, Japan’s cervical cancer prevention program has been inadequate. In 2016, the human papillomavirus vaccination and Pap smear rates were only 0.3% and 34%, respectively^2–4^. Consequently, the number of cervical cancer cases increased gradually from approximately 13,075 in 2000 to 34,782 in 2018, showing a > 2.5-fold increase^5,6^. Similarly, the number of deaths has increased over the years, from 2393 in 2000 to approximately 2921 in 2019^7^. In developing countries, the prevention program has not been fully implemented; thus, cervical cancer remains a life-threatening disease.

Cervical cancer is usually diagnosed based on the combination of pelvic examination, colposcopy, and radiological findings. Diagnostic conization is one of the options for more accurately evaluating the spread of the disease, particularly in the early stage. According to the National Comprehensive Cancer Network (NCCN) guidelines (Version 4.2019 Cervical Cancer), “cone biopsy (i.e., conization) is recommended if the cervical biopsy is inadequate to define invasiveness or if accurate assessment of microinvasive disease is required” (MS-4)^8^. Subsequently, the treatment method is determined based on the stage and pathological findings of conization. Radical hysterectomy, concurrent chemoradiation (CCRT), and radiation therapy (RT) are major treatment options for cervical cancer. In the case of radical hysterectomy, it is necessary to evaluate whether pelvic lymph node dissection should be performed because adverse events such as lymphedema can occur. Therefore, it is highly essential that accurate preoperative risk assessment of lymph node metastasis be performed. As reported in several studies, LVSI is a risk factor for lymph node metastasis; therefore, diagnostic conization can be useful for risk assessment^9–13^. However, there is a concern regarding excessive conization for locally advanced cases, which may include excising the tumor itself. Clinically, rapid disease progression is sometimes observed in patients with positive margins at conization, but the details are unclear.

The aim of this study was to evaluate the clinical impact of cervical conization with positive margins; therefore, the rate of positive lymph nodes, overall survival (OS), and progression-free survival (PFS) were evaluated as endpoints.

## Patients and methods

**Figure 1:**
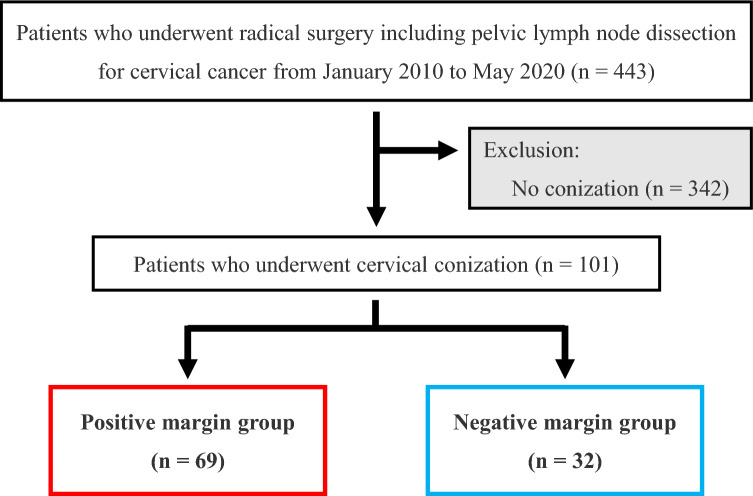
Flowchart of patient selection. Of the 443 patients who underwent radical surgery, including pelvic lymph node dissection for cervical cancer from January 2010 to May 2020, we excluded 342 patients without conization and included 101 patients with conization. Of the 101 patients, 69 had positive margins at conization and 32 had negative margins.

We retrospectively reviewed the records of 443 patients with cervical cancer who underwent radical surgery at Nagoya University Hospital (Nagoya, Japan) from January 2010 to May 2020. Several gynecologic oncologists of the multidisciplinary tumor board determined the treatment strategies for each patient and performed diagnostic conization when biopsy findings were insufficient. In accordance with the Japanese guidelines, in cases of operable early-stage cancer, radical surgery and pelvic lymph node dissection were performed and postoperative adjuvant therapy of CCRT or RT was considered based on the pathological findings. Conversely, for stage IIB (FIGO 2008) that are difficult to operate or for locally advanced cases such as stage III and IVA, CCRT is initially chosen. Regarding postoperative adjuvant therapy, CCRT was performed for the high-risk group of postoperative recurrence that met the criteria of positive parametrium invasion or positive pelvic lymph node metastasis in accordance with the guidelines. For the intermediate-risk group that met one of the following criteria, i.e., positive LVSI, deep cervical stromal invasion, or large cervical mass, RT or CCRT was selected after careful consideration of the number and degree of the risk factors. Of the 443 patients, 101 patients underwent conization followed by radical surgery, excluding 342 patients without conization (Fig. 1). Based on the pathological findings of conization, we classified the patients into positive margin (69 patients) and negative margin (32 patients) groups. We investigated the clinical information and compared the clinical outcomes between the two groups, including age, FIGO stage, histological type, tumor size at conization, stromal invasion at conization, LVSI at conization, LVSI at radical surgery, pelvic lymph node metastasis, adjuvant therapy, recurrence, and outcome. This study was approved by the Ethics Committee of Nagoya University (Approval No. 2019-0106, Nagoya University Graduate School of Medicine, Nagoya, Japan). All methods were performed in accordance with the relevant guidelines and regulations as well as in compliance with the requirements of the Declaration of Helsinki. Furthermore, informed consent was obtained from all participants.

Statistical analyses were performed using SPSS version 27 (IBM Corp., Armonk, NY). Mann–Whitney U test and chi-squared tests were used for comparing the two groups. OS was defined as the time from the primary therapy to all-cause mortality. PFS was the time from primary therapy to tumor progression, recurrence, and all-cause mortality. Kaplan–Meier curves were used for the analysis of OS and PFS, whereas the log-rank test was used to compare the survival curves. A *p* value of < 0.05 was considered statistically significant.

## Results

Patients’ characteristics are presented in Table 1. Age, stage, and histology were well balanced between the positive and negative margin groups. In addition, the tumor size and LVSI positivity were both not significantly different between the two groups (*p* = 0.296 and 0.417, respectively). The degree of stromal invasion, which was divided into three categories (superficial, middle, and deep) according to the NCCN guidelines, also showed no statistically significant difference between the two groups ((*p* = 0.359).

**Table 1:** Characteristics of 101 patients.

	Positive margin group (n = 69)	Negative margin group (n = 32)	*p* value
**Age (years)**			0.242
Median (range)	34 (25–59)	37 (23–68)	
**Stage (FIGO 2008)**			0.379
IA1	3 (4.3%)	1 (3.1%)	
IA2	7 (10.1%)	5 (15.6%)	
IB1	56 (81.2%)	26 (81.3%)	
IB2	1 (1.4%)	0	
IIA1	2 (2.9%)	0	
**Histological type**			0.377
SCC	51 (73.9%)	26 (81.3%)	
AC	12 (17.4%)	5 (15.6%)	
ASC	6 (8.7%)	1 (3.1%)	
**Tumor size (mm)**			0.296
Median (range)	11 (2–21)	10.5 (4–20)	
**Stromal invasion**			0.359
Superficial	50 (83.3%)	28 (90.3%)	
Middle	9 (15%)	3 (9.7%)	
Deep	1 (1.67%)	0	
**LVSI at conization**			0.417
Positive	34 (49.3%)	13 (40.6%)	
Negative	35 (50.7%)	19 (59.4%)	

*SCC* squamous cell carcinoma, *AC* adenocarcinoma, *ASC* adenosquamous carcinoma.

*p* value of < 0.05 was considered statistically significant.

First, we investigated whether the positive margins at conization contributed to cancer progression. The LVSI positive rate at radical surgery was significantly higher in the positive margin group than in the negative margin group (21.7% and 3.1%, respectively; *p* = 0.017; Table 2). Similarly, pelvic lymph node metastasis increased in the positive margin group; however, there was no significant difference between the groups (positive and negative margin groups, 11.6% and 3.1%, respectively; *p* = 0.155). In addition, patients with stage IB1 squamous cell carcinoma comprise the main subpopulation or focused patients of this cohort study. Similarly, LVSI at radical surgery was also significantly higher in patients in the positive margin group than in those in the negative margin group (27.3% and 0%, respectively; *p* = 0.005; Table 3). Moreover, a high frequency of lymph node metastasis was observed in the positive margin group, with no significant difference (positive and negative margin groups, 11.4% and 4.8%, respectively; *p* = 0.362).

**Table 2:** The association between a positive margin at conization and metastatic potential in all patients.

	Positive margin group (n = 69)	Negative margin group (n = 32)	*p* value
**LVSI at radical surgery**			0.017
Positive	15 (21.7%)	1 (3.1%)	
Negative	54 (78.3%)	31 (96.9%)	
**Lymph node metastasis**			0.155
Positive	8 (11.6%)	1 (3.1%)	
Negative	61 (88.4%)	31 (96.9%)	

*LVSI* lymphovascular space invasion.

*p* value of < 0.05 was considered statistically significant.

**Table 3:** The association between a positive margin at conization and metastatic potential in stage IB1 squamous cell carcinoma.

	Positive margin group (n = 44)	Negative margin group (n = 21)	*p* value
**LVSI at radical surgery**			0.005
Positive	12 (27.3%)	0 (0%)	
Negative	32 (72.7%)	21 (100%)	
**Lymph node metastasis**			0.362
Positive	5 (11.4%)	1 (4.8%)	
Negative	39 (88.6%)	20 (95.2%)	

*LVSI* lymphovascular space invasion.

*p* value of < 0.05 was considered statistically significant.

**Figure 2:**
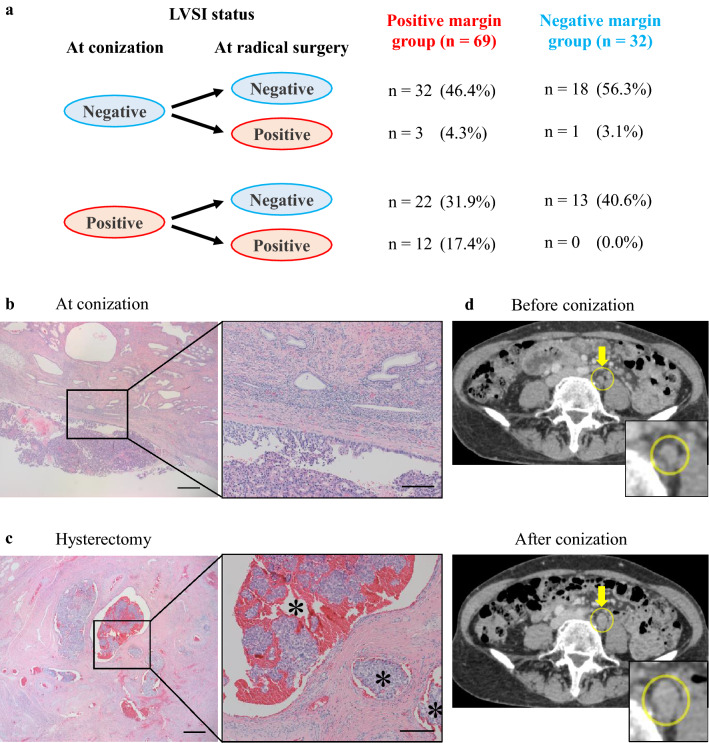
LVSI status at conization and at radical surgery. **(a)** The relationship between LVSI at conization and at radical surgery. **(b,c)** Representative histological images. Asterisks indicate tumors in vessels (LVSI positive). The scale bars: left; 50 µm and right; 20 µm. **(d)** Representative computed tomography images of the case before/after conization.

Subsequently, we evaluated the differences between the LVSI status at conization and at radical surgery (Fig. 2a). The LVSI statuses at conization and radical surgery were consistent in most patients. However, negative LVSI at conization became positive at radical surgery in four cases, three of which were in the positive margin group. Detailed clinical information about one of the cases is shown in Fig. 2b–d. A patient underwent diagnostic conization because both Pap smear test and biopsy were negative, despite suspicions of cervical cancer by the transvaginal ultrasound. In conization, the tumor was resected to the maximum extent possible by cutting through the tumor wall. Hematoxylin–eosin staining showed adenocarcinoma with a negative LVSI and positive surgical margin on the uterine side (Fig. 2b). However, after radical surgery, LVSI turned to be highly positive and was diagnosed as pT2a1 (Fig. 2c). Moreover, the obvious enlargement of the left common iliac lymph node was identified using computed tomography, although there was no swelling before conization (Fig. 2d), and the lymph node was pathologically positive for metastasis.

**Figure 3:**
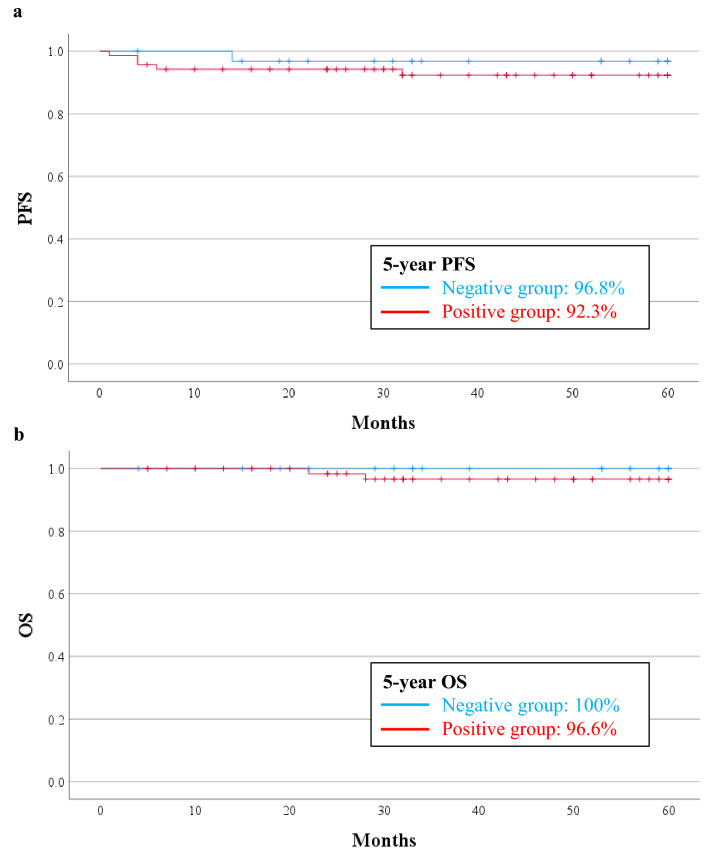
Prognosis of the patients. Kaplan–Meier curves showing **(a)** PFS and **(b)** OS stratified by the positive and negative margin groups. Survival curves were compared using the log-rank test, but there were no significant differences in PFS and OS between the two groups.

Finally, we analyzed the PFS and OS of the positive and negative margin groups. The median follow-up period was 56 months (range 4–124 months). The 5-year PFS rates in the positive and negative margin groups were 92.3% and 96.8%, respectively (Fig. 3a). The 5-year OS rates in the positive and negative margin groups were 96.6% and 100%, respectively (Fig. 3b). Kaplan–Meier curves indicated that there were no statistically significant differences between the two groups in PFS (*p* = 0.200; log-rank test) and OS (*p* = 0.332; log-rank test).

## Discussion

According to the Japanese and the NCCN Clinical Practice Guidelines in Oncology, diagnostic conization is an important method for determining the exact stage of cancer and most accurate treatment strategy for patients with cervical cancer^8,14,15^. However, as depicted by the results of this study, there is a potential risk of cutting a tumor in some cases, resulting in a positive margin at conization. To date, there is no consensus on whether diagnostic conization is appropriate even when positive margins are predicted. Therefore, we evaluated the association between diagnostic conization with positive margins and patient outcomes, including lymph node metastasis, in uterine cervical cancer.

The basic principles of oncologic surgery are careful tumor manipulation, resection with tumor-free margins, and avoidance of tumor spillage^16^. It has been suggested that these principles are associated with prognosis, examples of which are considered in cervical cancer. Patients who underwent minimally invasive radical hysterectomy could have shorter survival than those who underwent conventional abdominal radical hysterectomy. A possible explanation is the use of an intrauterine manipulator in minimally invasive surgery, which might increase the intrauterine pressure and spread the cancer cells into the lymphovascular space^16–19^. However, other reports showed that the use of manipulators is not associated with worse prognosis, and its clinical significance remains controversial^20,21^. Thus, the basic principles in oncologic surgery are important; although they are not the absolute factors, there could be other factors that have a great influence.

In addition, diagnostic conization does not meet these principles in case of a positive margin. The risk of positive margins at conization was associated with several factors, such as menopausal status, grade and size of the disease, devices used for conization, and the purpose of conization (diagnostic or therapeutic)^22–24^. Previous studies have demonstrated that a positive margin at conization would mean residual disease; however, it was not associated with parametrial invasion at the time of hysterectomy^25–27^. This study showed that positive margins may increase the rate of LVSI positivity in hysterectomy samples, although there was no statistical significance regarding pelvic lymph node metastasis and no significant difference in PFS and OS between the positive and negative margin groups. Consistent with the results of this study, a recent report also indicated that there were no significant differences in lymph node metastasis, LVSI positivity, recurrence, and death between patients with positive and negative margins^28^. Therefore, the prognostic impact of a positive margin at conization was considered limited.

However, in some cases, obvious lymph node enlargement and strong LVSI positivity can occur after diagnostic conization with a positive margin. Nonetheless, lymph node metastasis is one of the worst prognostic factors in cervical cancer^29,30^. Therefore, regardless of the lack of statistical significance, clinicians should keep in mind that there are cases with rapid disease progression after conization. Moreover, owing to this relatively small-scale retrospective study, it was difficult to statistically evaluate such rare cases. Therefore, further studies are warranted to evaluate the potential risk of a positive margin at conization.

The number of LVSIs found in radical surgery specimens is extremely low compared with that in cervical conization specimens. There are approximately 30–40% of cases that are positive at the time of conization but negative in the hysterectomy specimen. Most cases are early-stage cancers and the tumors are not large; even if LVSI is positive at the time of conization, there may be cases in which LVSI is negative in the specimen at the time of radical surgery because the main tumor is removed. Conversely, it may be related to pathological methods such as the small number of sections to be evaluated at the time of radical surgery. It is also possible that more positive LVSIs can be found if more sections are prepared. The above is just one example, but a large number of confounding factors is one of the limitations.

In conclusion, this study showed that there was no significant difference in lymph node metastasis rates or patient prognosis between the positive and negative margin groups. The association between cervical conization with positive margins and subsequent cancer progression was not demonstrated. Therefore, diagnostic conization is one of the suitable options for early-stage cervical cancer detection.
